# The Effects of Counting the Stride Numbers on the Parkinsonian Gait: Suggesting a Possible Reason for Dual Task Interference

**DOI:** 10.32598/bcn.9.10.245

**Published:** 2019-05-01

**Authors:** Yashar Sarbaz, Hakimeh Pourakbari

**Affiliations:** 1. Department of Mechatronics Engineering, School of Engineering Emerging Technologies, University of Tabriz, Tabriz, Iran.

**Keywords:** Parkinson’s Disease, Early diagnosis, Internal biological clock, Dual-task

## Abstract

**Introduction:**

Parkinson Disease (PD) is a degenerative and progressive disorder of the central nervous system. It results from degeneration of Substantia Nigra pars compacta (SNc) of the Basal Ganglia (BG). Gait disturbances in PD patients generally get worse over time. The underlying mechanism of gait disturbances in PD has not been elucidated yet.

**Methods:**

In this study, we tried to analyze changes in walking performance under both single- and dual-task conditions in people with PD compared to healthy subjects. To this end, the participants’ trunk acceleration signals were recorded under dual-task (counting the stride number while walking) and single-task (walking without performing any other secondary tasks) conditions.

**Results:**

The healthy subjects counted the number of their strides correctly; however, 85% of the patients made glaring errors in counting. Then variances of Stride Time Interval (STI) signals were calculated for each participant. STI signals of the patients had a higher variance than that of the healthy subjects in the dual-task condition. Separating the two groups in a dual-task condition is easier. Therefore, we think that the disease sate can be detected in early stages. It is thought that counting is performed independent of walking.

**Conclusion:**

PD affects the function of BG that leads to motor timing dysfunction. So, it seems that timing in motor tasks is disrupted while timing in cognitive tasks is not. Therefore, perhaps inconsistency between the two clocks (motor-tasks and cognitive-tasks clocks) is the main cause of dual-task interference in patients’ gait.

## Highlights

- The body’s biological clock drives various physical and mental activities throughout the body.
- Parkinson disease disrupts the body clock that leads to motor timing dysfunction.
- Presumably, there are two distinctive body clocks (motor tasks and cognitive tasks clocks) that lose their synchrony in disease states.
- The inconsistency between cognitive and motor tasks clocks discriminates between healthy subjects and patients.
- This inconsistency can be used as an adjacent theory in the diagnosis of the Parkinson disease in its early stages.

## Plain Language Summary

Parkinson Disease (PD) is a degenerative disorder of the Central Nervous System (CNS) that mainly affects the motor system. Gait disturbances are common motor disorders in PD. According to some researchers, the body’s internal biological clock drives various physical and mental activities throughout the body and PD affects the functions of the body clock that leads to motor timing dysfunction. Since the cognitive abilities of the brain are less affected by PD, we can assume that there are two clocks (motor tasks clock and cognitive tasks clock) that lose their synchrony in disease states. As the disease progresses, the inconsistency between these two clocks gets worse. It is postulated that this inconsistency is the main cause of gait disturbances. To test this hypothesis, we studied and compared PD patients and age-matched healthy subjects. They were instructed to walk and count the number of strides taken while walking. We observed that the healthy subjects counted the number of their strides correctly, whereas 85% of the patients made apparent errors in counting the stride numbers. It seems that counting (that is a cognitive task) is performed independently of walking (that is a motor task). Based on our study results, we think that PD can be detected in its early stages by this method

## 1. Introduction

Parkinson’s Disease (PD) is a degenerative and progressive disorder of the Central Nervous System (CNS), which results from degeneration of Substantia Nigra pars compacta (SNc) of the Basal Ganglia (BG). The real cause of this destruction is still unknown with no definitive treatment. Unfortunately, there are not enough diagnostic or effective screening tools in order to identify the patients in early stages of PD. In fact, approximately about 50% to 80% of SNc neurons have already been damaged, when PD has been diagnosed. It is believed that early diagnosis and treatment of PD can slow down the progression and postpone the severe stages of the disease (Aarsland, Brønnick, Larsen, Tysnes & Alves 2009).

PD mainly affects the motor system. Gait disturbances in PD patients are due to motor system disorders that generally get worse over time. These abnormalities are characterized by bradykinesia, muscle rigidity, asymmetry of the left and right parts of the body, abnormal rhythmicity, decreased force generation, and abnormal scaling of pace length. PD negatively affects gait parameters such as step length, stride length, speed, cadence, double support time and so on (Brown, de Bruin, Doan, Suchowersky & Hu, 2009). Since postural imbalance and rigidity are cardinal symptoms of PD and are shown in gait disorders, gait analysis may be crucial for the PD diagnosis. It is important to point out that the underlying mechanism of gait disturbances in PD has not yet been fully elucidated.

Several studies have been conducted to analyze the gait disturbances in PD patients since 1980s (Carson, Butcher & Carson 1980). Sarbaz et al. studied the frequency variations of Stride Time Intervals in people with PD (De Nunzio, Grasso, Nardone, Godi & Schieppati, 2010). Also, some studies have explored the chaotic features of gait in PD and proposed some quantitative models for gait disturbances that can be effective in early diagnosis of the disease (Factor & Weiner, 2007; Foltynie, Brayne, Robbins & Barker, 2004; Hausdorff, 2007).

Hausdorff (2009) investigated gait disturbances in people with PD and found statistical differences in stride behavior between healthy subjects and patients with PD. Claimed that rhythmic auditory stimulation can improve the speed of gait, swing time, and stride length (Hausdorff, Lowenthal, Herman, Gruendlinger, Peretz, & Giladi, 2007).

It was also shown that dual-tasking is impaired in patients with PD (Jones et al.,2011; Joundi, Brittain, Green, Aziz & Jenkinson 2012; Kelly, Eusterbrock, & Shumway-Cook; 2012). Extensive studies have investigated dual-task performance during walking in patients with PD (Knutsson 1972; Lord, Howe, Greenland, Simpson, & Rochester, 2011; Meck, Penney & Pouthas, 2008). O’Shea et al.(2002), compared the walking patterns of 15 patients with PD and 15 healthy subjects under single-task and two dual-task (a cognitive and a motor secondary task) conditions.

The participants were asked to walk at their self-selected speed in each condition. For the secondary motor and cognitive tasks, coin transference and digit subtraction were implemented. The results revealed that gait patterns were exacerbated under both dual-task conditions (Mera et al., 2013).

In 2014, Stegemöller’s research group examined the impact of cognitive performance on gait spatiotemporal parameters. In this study, 35 patients with PD participated. They walked barefoot along a 12-m walkway at their preferred speed while simultaneously counting backward from a 3-digit number by 3’s. Based on the results, dual task reduced gait speed and stride length and also led to an increment of gait variability (Morris, Iansek, Smithson & Huxham, 2000). Similar studies have found the same results, too (Okuno et al., 2008; O’Shea, Morris & Iansek, 2002).

Based on the previous studies, gait is disturbed during dual-task walking in people with PD. However, no significant attention has been paid to the cause of dual-task interference during gait. In our previous study (Perbal et al., 2005), we tried to present a clinical gait-test protocol and its potential value for separating patients with PD from healthy subjects. In the current study, efforts were made to analyze gait disturbances under single-task and dual-task conditions. Finally, based on the clinical observations, an explanation will be proposed for the cause of dual-task interference during gait.

## 2. Methods

In our previous study, we designed a protocol for gait data acquisition, in which the participants walked in an “8-shaped path” for 3 minutes and counted the number of strides taken while walking, and we recorded their acceleration data. The study included 7 healthy subjects and 7 patients with PD (Perbal et al., 2005). As larger sample size yields more reliable results with greater precision, we increased the number of participants and repeated the study. Also, in order to understand the effects of the dual-task intervention on gait performance of the patients with PD, the participants’ trunk acceleration signals were recorded under single-task (walking without counting) condition.

The current study included 20 male patients with PD, able to walk without any help and 18 age-matched male healthy subjects. The patients should not take any drug 8 hours before initiation of the test. This helped us to fully determine the effects of the disease on the patients’ behavior and prevented drug effects on the symptoms. Falling or Freezing of Gait (FOG) was not present in the patients. The stage of gait disturbances in the studied patients was between 1 and 3. The severity of illness was determined by an expert physician, according to Hoehn and Yahr scale (Aarsland et al., 2009).

Table 1 presents the characteristics of the participants. The designed device for measuring the Stride Time Intervals (STI) included a three-axis acceleration sensor (ADXL303) that could measure the trunk acceleration of the participants (Perbal et al., 2005; Plotnik, Dagan, Gurevich, Giladi & Hausdorff, 2011). The accelerometer outputs were digitized at a sampling rate of 256 Hz. Then, an AVR microcontroller sent the acceleration data to a wireless RF transmitter. The RF receiver was connected to a PC to relay the recorded signals to the PC via AVR and FT232 chip. In order to record the acceleration signals, the transmitting part of the device was placed on the participant’s waist, in a midline position.

**Table 1.** The characteristics of the participants

**Characteristics**	**Patients (n=20)**	**Healthy Subjects (n=18)**
Age, y	59	55
Hoehn and Yahr stage	1.7±0.6	-

The data are presented as Mean±SD.

The participants were instructed to walk at their normal speed in an “8-shaped path” (between 2 chairs with a distance of 2 meters from each other) for 3 minutes and count the number of strides taken while walking (dual-task condition). An observer also counted the participant’s stride number, simultaneously. They did the same test after resting (at least 3 minutes) without counting or performing any other cognitive tasks (single-task condition). Comparing the STI signals of some healthy subjects (under-single and dual-task condition) showed they had similar stride behavior under both conditions. Therefore, the rest of the healthy subjects did not perform the second test.

MATLAB was used to process the recorded signals, in order to extract STI signals, by detecting the peaks of the acceleration raw signals. Indeed, the time between every other peak was considered as one Stride Time Interval. For example, the time between the first and the third peak was the first stride of the left foot and the time between the second and the fourth peak was the first stride length of the right foot. The transmitter part of the device was placed on the participant’s waist, in a midline position. It should be noted that there was no need to find the exact position of all participants’ bodies to place the transmitter. Since time intervals between two consecutive peaks of the acceleration signals are considered, minor positioning differences between the subjects’ bodies do not affect the acceleration signals.

By this method, all participants’ STI (right foot and left foot) signals were extracted. The patients’ signals showed great stride-to-stride fluctuations, while small variations were observed in healthy subjects’ STI signals. After evaluating the behavior of STI signals, it has been realized that there may be differences between the variances of STI signals of the patients and healthy subjects. Therefore, the variances of STI for the left and right foot of each subject were calculated.

After extracting variances of STI signals, we intended to know whether or not the differences between healthy subjects and PD patients are significant. Thus, we used the following statistical analyses. To test whether these distributions (healthy subjects and patients) are significantly different from each other, we performed a two-sample Kolmogorov-Smirnov test on each pair of distributions. Thereafter, 1-way Analysis of Variance (ANOVA) was applied to compare STI variances between healthy subjects and PD patients. This was done in order to determine if there were any significant differences between the mean values of the two groups (healthy subjects and PD patients). In the following, we used the Tukey-Kramer method for doing post hoc comparisons of healthy subjects’ and patients’ mean results to more precisely determine the differences between the groups.

## 3. Results

At this stage, the number of strides reported by the participants was compared and the observer’s. The healthy subjects counted the number of their strides correctly (error of less than 1%). Whereas, 85% of the patients made glaring errors in counting the stride numbers. Where there was more than 20% difference between the reported number by the participant and the observer, this was recorded as a glaring error.

**Figure 1.**
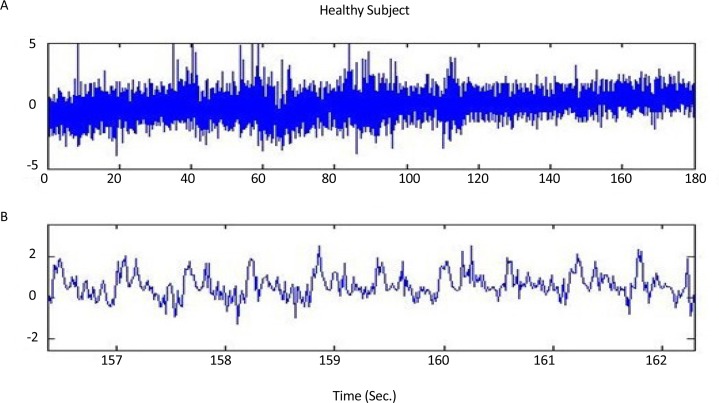
Raw acceleration signal over time a. A healthy subject’s raw acceleration signal over time; b. A healthy subject’s raw acceleration signal over time in a shorter time scale.

Figure 1 a, shows a healthy subject’s raw (without any filtering) acceleration signal over time under the dual-task condition. Figure 1 b, is presented at a shorter time scale in order to more clearly show the acceleration signal behavior.

**Figure 2.**
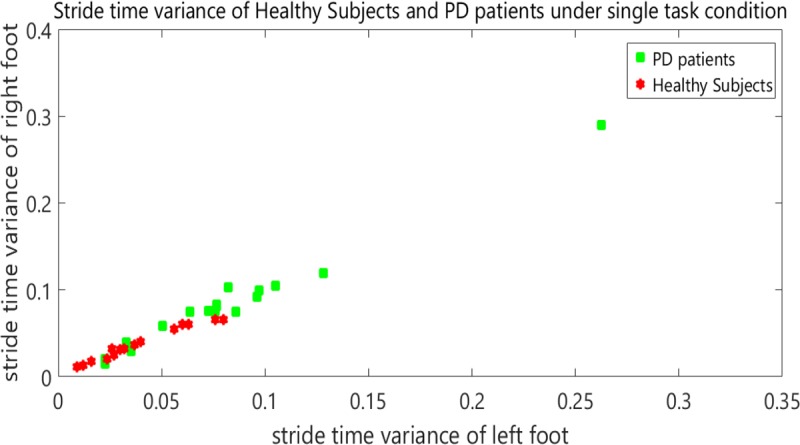
STI variances for healthy subjects and patients with PD under single-task condition™

**Figure 3.**
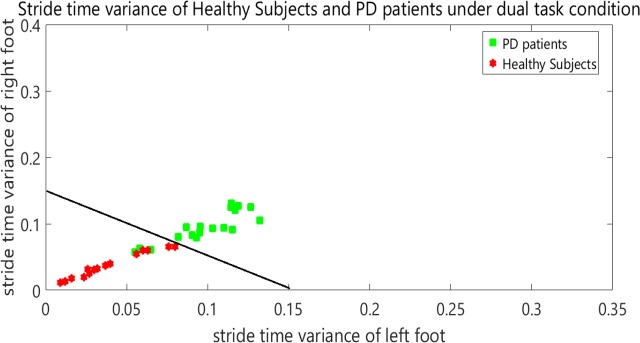
STI variances for healthy subjects and patients with PD under dual-task condition

Figure 2 shows the STI variances of healthy subjects, along with STI signals of patients under single-task condition. The vertical axis shows the right STI variance and the horizontal axis shows the left STI variance. Figure 3 represents the STI variances of healthy subjects, along with STI signals of patients under dual-task condition.

In order to separate healthy subjects from patients with PD (under dual-task condition), the variance space was divided into two distinct (healthy and diseased) sections in the Figure 3 by a straight line with the following equation:

y=f(x)=−x+0.15

It is worth mentioning that the selected classifier in this study is a linear space divider. This has divided the space of the healthy and diseased state with the presented relation. This classifier is simple and elementary. It has been presented only to show significant differences between the groups. It is clear that more powerful classifiers such as artificial neural networks or support vector machines will increase the accuracy of this screening. Table 2 presents the results of the classifier.

**Table 2.** Accuracy, sensitivity and specificity values of the classifier

**Measures of Classifier**	**Accuracy**	**Specificity**	**Sensitivity**
Result	92.10%	100%	85%

**Figure 4.**
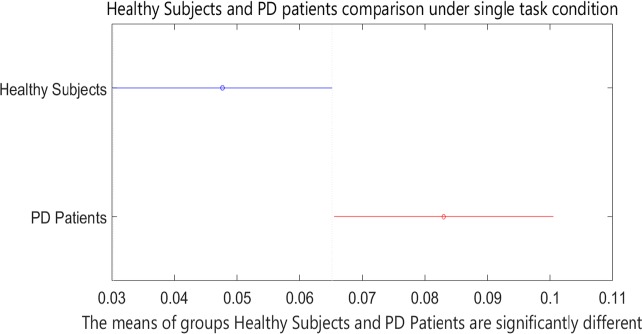
Post hoc analysis results for healthy subjects and patients with PD under single-task condition

**Figure 5.**
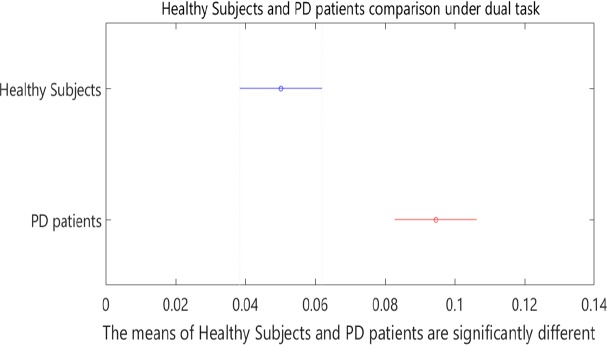
Post hoc analysis results for healthy subjects and patients with PD under dual-task condition

Based on the two-sample Kolmogorov-Smirnov test, the healthy subjects and patients with PD were found to be significantly different from each other at the significance level of α=0.001. Table 3 presents P values in 1-way ANOVA test. Post hoc analysis results are shown in Figures 4 and 5.

**Table 3.** One-way ANOVA P-values for STI variances of healthy subjects and patients with PD

**P for STI Variances**	
Healthy subjects and patients with PD (single task condition)	0.048
Healthy subjects and patients with PD (dual task condition)	<0.01

**Figure 6.**
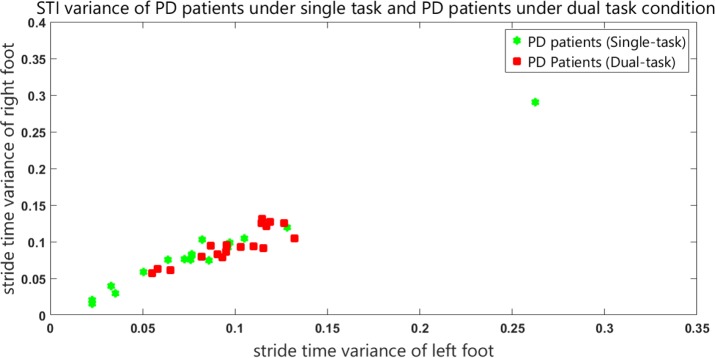
STI variances for patients with PD under the single-task condition and patients with PD under dual-task condition

**Figure 7.**
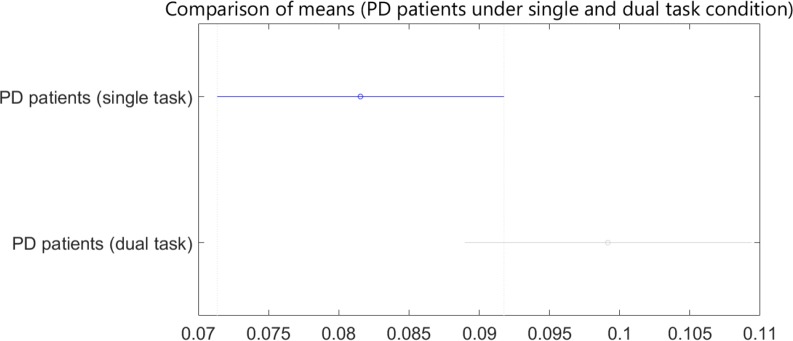
Post hoc analysis results for patients with PD under single-task and patients with PD under dual-task condition

As the results demonstrated, dual task revealed statistically significant differences between the healthy subjects and patients with PD. In the final stage, the patients’ STI signals under single-task and dual-task conditions were compared. Figure 6 shows the STI variances of the patients with PD under the single-task condition, along with STI signals of patients with PD under dual-task condition. ANOVA results had a P value of 0.08. Also, post hoc analysis result is shown in Figure 7.

Of course, we didn’t expect to perform the secondary task that could precisely screen patients under dual-task and single-task conditions. Counting the stride numbers exacerbates gait disturbances. It should be noted if healthy subjects and patients with PD are compared under single- and dual-task conditions, significant differences between healthy subjects and patients under the dual-task condition are observed when compared to the single-task condition, as seen in the post hoc results (Figure 5). As a result, performing such a cognitive task makes screening of the patients with PD simpler and more effective.

## 4. Discussion

Walking is a highly complex motor process that is regulated by the brain. Much remains to be elucidated concerning the neural mechanisms underlying movement control in the brain. CNS diseases can cause problems with the control of movements. Such diseases to some extent help us better understand the movement control mechanisms. Each disease affects particular areas of the brain; hence, destruction of that particular area reveals its role in the control of the movement.

PD is a degenerative disorder of the CNS that mainly affects the motor system. Gait disturbances in PD are due to motor system disorders that generally get worse over time. The underlying mechanism of gait disturbances in PD has not been determined yet. It is believed that early diagnosis and treatment of PD can slow the progression of the disease, and as a result, can postpone the severe stages of the disease. In the present study, attempts were made to evaluate the behavior of people with PD and propose an explanation for the cause of gait disturbances in order to achieve a better understanding of human movement control mechanisms. If the internal clock function will be specified, we hope this idea be helpful in early diagnosis of PD.

We proposed a new protocol for gait data acquisition in an “8-shaped path” in our previous study (Pradhan et al., 2010). The participants counted their stride number while walking. The results clearly showed its potential value for separating patients with PD from healthy subjects. In the current study, we tried to increase the reliability of the results by increasing the sample size (20 patients with PD and 18 healthy subjects) and investigating the effects of dual-task interference on gait performance. To this end, in this study, the healthy subjects and patients with PD were asked to walk in an “8-shaped path” for 3 minutes and count the number of strides taken while walking. The patients did the same test after resting, without counting or performing any other cognitive tasks.

Then, the participants’ trunk acceleration signals were recorded while they walked. STI signals were extracted from the acceleration signals. Next, variances of STI signals were calculated and considered as features. Statistical analyses showed that counting the stride number improves the reliability of screening patients out of healthy subjects; however, there were no significant differences between the patients under dual-task and secondary task conditions. It means that counting does not necessarily alter gait performances in patients but affects the variance and increases the differences between patients and healthy subjects. Therefore, it is helpful in separating healthy subjects from patients. An interesting point is that the healthy subjects counted the number of their strides correctly; however, most of the patients made glaring errors in counting the stride numbers. To our knowledge, few studies have been conducted on this topic. It seems that this study may describe the reason for dual-task interference in the patients.

Here, the main unanswered questions are as follows: what the causes of dual-task interference in the patients are, why patients manifested worse gait performance under the dual-task condition, and why patients make errors in counting the strides number.

Based on the clinical observations, there is a significant difference between cognitive performance (counting the number of strides) of the healthy subjects and patients with PD. The body’s internal biological clock influences various physical and mental activities throughout the body. It also facilitates synchronization of movements and stabilizes walking. Various disorders may affect performances of the internal clock, including motor control, cognitive performance, alertness, mental health, and metabolism (Pradhan et al., 2010).

Joundi et al. showed that Basal Ganglia (BG) had a major role in regulating motor timing (Proud & Morris, 2010). BG has an important role in the production of rhythmically repetitive movements. The internal clock is tuned on by BG during finger tapping performance (Sarbaz & Pourakbari, 2015). As already proved, PD affects the BG. The results of Perbal et al. study shows that dopaminergic dysfunction slows down the rate of the body’s internal (biological) clock (Sarbaz et al., 2012).

Hausdroff et al. (2009), suggested that impairment of “internal clock” function lead to gait disturbances (Sarbaz, Gharibzadeh, Soltanzadeh, & Towhidkhah, 2013). As mentioned before, the main reason for synchronization of all actions of the body seems to be the internal biological clock. Indeed, all parts of the CNS regulate their performances timing according to the internal clock in a healthy state. BG has an important role in planning and regulating motor tasks of the body. PD affects BG and, as a result, timing in motor tasks is disrupted in people with PD.

We propose that another clock is established for motor tasks that we call it “motor tasks clock”. Also, we can consider a “cognitive task clock” for cognitive tasks. These two clocks are concurrent with the internal clock and synchronized in a healthy state. Because PD affects BG, it cannot regulate its activity based on the internal clock. However, cognitive task clock still coincides with the internal clock. Therefore, the synchronization between these two clocks is disrupted in the diseased state. That is why the patients made errors in counting the stride number (cognitive task) while walking (motor task). Indeed, these two tasks are regulated according to two inconsistent timing.

A few studies have been conducted to investigate the internal clock role in regulating the body’s activities that may confirm this idea. It should be noted that to the best of our knowledge such an idea (inconsistency between the two clocks) has not been presented so far. It should be noted that the role of BG on motor tasks timing has been studied before, but its role on cognitive tasks timing and also the coordination between cognitive and motor tasks has not been studied much. An inconsistent relationship between the timing of a motor task and the timing of a cognitive task may appear in the early stages of the PD (Sarbaz, Gharibzadeh, Towhidkhah, Banaie, & Jafari, 2011; Sarbaz et al., 2012).

It is possible that all movement disorders in PD are not caused solely by damage to the motor areas of the CNS (Sarbaz, Towhidkhah, Jafari & Gharibzadeh 2012). Perhaps this timing inconsistency has a larger role in the appearance of movement disorders (Stegemöller et al., 2014). Therefore, based on this idea, desynchronization is the result of creating two different timing (motor-task timing and cognitive-task timing). This is due to BG damage and motor timing disruption.

Based on the presented idea, the two clocks don’t show coinciding component periods under the diseased state. Therefore, it is possible to develop a new theory about disease disorders appearance. Moreover, functional performances of the disease rehabilitation may be explained. For example, some studies have shown the effectiveness of vibratory stimulation on gait in PD patients (Wild et al., 2013). Additionally, Deep Brain Stimulation (DBS) can improve movement disorders of PD (Wylie et al., 2012).

It is possible that regular electrical impulses create a reference clock that coordinates cognitive and motor clocks. In the case of synchronization between the two clocks and tuning of the synchronization with an external resource clock (DBS), the time difference between the two clocks reduces and as a result the degree of gait disturbances decreases.

The results of this study demonstrate an increase in dual-task gait disturbances. This finding may be appropriate for the detection of PD in its earlier stages. However, it seems that the main cause of the dual-task interference during gait is related to disharmony between the motor-task clock and cognitive-task clock. One of the major findings of this study is that any cognitive activity that involves the brain besides motor activity will lead to increased effectiveness of screening of patients with PD. It should be noted that the more the brain is involved during motor activities, the more accurate will be the screening procedure.

## References

[B1] Aarsland D. Brønnick K. Larsen J. P. Tysnes O. B. Alves G. Norwegian ParkWest Study Group (2009). Cognitive impairment in incident, untreated Parkinson’s Disease: The Norwegian ParkWest Study. Neurology, 72(13), 1121–6. [DOI:10.1212/01.wnl.0000338632.00552.cb] [PMID ] 19020293

[B2] Brown L. A. de Bruin N. Doan J. B. Suchowersky O. Hu B. (2009). Novel challenges to gait in Parkinson’s disease: The effect of concurrent music in single-and dual-task contexts. Archives of Physical Medicine and Rehabilitation, 90(9), 1578–83. [DOI:10.1016/j.apmr.2009.03.009] [PMID ] 19735787

[B3] Carson R. C. Butcher J. N. Coleman J. C. (1988). Abnormal psychology and modern life. Glenview, Illinois: Scott Foresman.

[B4] De Nunzio A. M. Grasso M. Nardone A. Godi M. Schieppati M. (2010). Alternate rhythmic vibratory stimulation of trunk muscles affects walking cadence and velocity in Parkinson’s disease. Clinical Neurophysiology, 121(2), 240–7. [DOI:10.1016/j.clinph.2009.10.018] [PMID ] 19955020

[B5] Factor S. A. Weiner W. J. (2007). Parkinson’s disease: Diagnosis & Clinical Management. New York: Demos Medical Publishing.

[B6] Foltynie T. Brayne C. E. Robbins T. W. Barker R. A. (2004). The cognitive ability of an incident cohort of Parkinson’s patients in the UK: The CamPaIGN study. Brain, 127(3), 550–60. [DOI:10.1093/brain/awh067] 14691062

[B7] Hausdorff J. M. (2007). Gait dynamics, fractals and falls: Finding meaning in the stride-to-stride fluctuations of human walking. Human Movement Science, 26(4), 555–89. [DOI:10.1016/j.humov.2007.05.003] [PMID ] [PMCID ] 17618701 PMC2267927

[B8] Hausdorff J. M. (2009). Gait dynamics in Parkinson’s disease: Common and distinct behavior among stride length, gait variability, and fractal-like scaling. Chaos: An Interdisciplinary. Journal of Nonlinear Science, 19(2), 026113 [DOI:10.1063/1.3147408] PMC2719464 19566273

[B9] Hausdorff J. M. Lowenthal J. Herman T. Gruendlinger L. Peretz C. Giladi N. (2007). Rhythmic auditory stimulation modulates gait variability in Parkinson’s disease. European Journal of Neuroscience, 26(8), 2369–75. [DOI:10.1111/j.1460-9568.2007.05810.x] [PMID ] 17953624

[B10] Jones C. R. G. Claassen D. Yu M. Spies J. R. Malone T. Dirnberger G. (2011). Modeling accuracy and variability of motor timing in treated and untreated Parkinson’s disease and healthy controls. Frontiers in Integrative Neuroscience, 5, 81 [DOI:10.1201/b11284] 22207839 PMC3245650

[B11] Joundi R. A. Brittain J. S. Green A. L. Aziz T. Z. Jenkinson N. (2012). High-frequency stimulation of the subthalamic nucleus selectively decreases central variance of rhythmic finger tapping in Parkinson’s disease. Neuropsychologia, 50(10), 2460–6. [DOI:10.1016/j.neuropsychologia.2012.06.017] [PMID ] 22749972

[B12] Kelly V. E. Eusterbrock A. J. Shumway-Cook A. (2012). A review of dual-task walking deficits in people with Parkinson’s disease: Motor and cognitive contributions, mechanisms, and clinical implications. Parkinson’s Disease, 2012, 918719 [DOI:10.1155/2012/918719] PMC3205740 22135764

[B13] Knutsson E. (1972). An analysis of Parkinsonian gait. Brain, 95(3), 475–86. [DOI:10.1093/brain/95.3.475] [PMID ] 4655275

[B14] Lord S. Howe T. Greenland J. Simpson L. Rochester L. (2011). Gait variability in older adults: A structured review of testing protocol and clinimetric properties. Gait & Posture, 34(4), 443–50. [DOI:10.1016/j.gaitpost.2011.07.010] [PMID ] 21920755

[B15] Meck W. H. Penney T. B. Pouthas V. (2008). Corticostriatal representation of time in animals and humans. Current Opinion in Neurobiology, 18(2), 145–52. [DOI:10.1016/j.conb.2008.08.002] [PMID ] 18708142

[B16] Mera T. O. Filipkowski D. E. Riley D. E. Whitney C. M. Walter B. L. Gunzler S. A. Giuffrida J. P. (2013). Quantitative analysis of gait and balance response to deep brain stimulation in Parkinson’s disease. Gait & Posture, 38(1), 109–14. [DOI:10.1016/j.gaitpost.2012.10.025] [PMID ] [PMCID ] 23218768 PMC3596454

[B17] Morris M. Iansek R. Smithson F. Huxham F. (2000). Postural instability in Parkinson’s disease: A comparison with and without a concurrent task. Gait & Posture, 12(3), 205–16. [DOI:10.1016/S0966-6362(00)00076-X] 11154931

[B18] Okuno R. Fujimoto S. Akazawa J. Yokoe M. Sakoda S. Akazawa K. (2008). Analysis of spatial temporal plantar pressure pattern during gait in Parkinson’s disease. Paper presented at the 30th Annual International Conference of the IEEE Engineering in Medicine and Biology Society, Vancouver, Canada, 20–25 August 2008 [DOI:10.1109/IEMBS.2008.4649519] 19163022

[B19] O’Shea S. Morris M. E. Iansek R. (2002). Dual task interference during gait in people with Parkinson’s Disease: Effects of motor versus cognitive secondary tasks. Physical Therapy, 82(9), 888–97. [PMID ] 12201803

[B20] Perbal S. Deweer B. Pillon B. Vidailhet M. Dubois B. Pouthas V. (2005). Effects of internal clock and memory disorders on duration reproductions and duration productions in patients with Parkinson’s disease. Brain and Cognition, 58(1), 35–48. [DOI:10.1016/j.bandc.2005.02.003] [PMID ] 15878725

[B21] Plotnik M. Dagan Y. Gurevich T. Giladi N. Hausdorff J. M. (2011). Effects of cognitive function on gait and dual tasking abilities in patients with Parkinson’s disease suffering from motor response fluctuations. Experimental Brain Research, 208(2), 169–79. [DOI:10.1007/s00221-010-2469-y] [PMID ] 21063692

[B22] Pradhan S. D. Brewer B. R. Carvell G. E. Sparto P. J. Delitto A. Matsuoka Y. (2010). Assessment of fine motor control in individuals with Parkinson’s disease using force tracking with a secondary cognitive task. Journal of Neurologic Physical Therapy, 34(1), 32–40. [DOI:10.1097/NPT.0b013e3181d055a6] [PMID ] 20212366

[B23] Proud E. L. Morris M. E. (2010). Skilled hand dexterity in Parkinson’s disease: Effects of adding a concurrent task. Archives of Physical Medicine and Rehabilitation, 91(5), 794–9. [DOI:10.1016/j.apmr.2010.01.008] [PMID ] 20434619

[B24] Sarbaz Y. Pourakbari H. (2015). A review of presented mathematical models in Parkinson’s disease: Black-and gray-box models. Medical & Biological Engineering & Computing, 54(6), 855–68. [DOI:10.1007/s11517-015-1401-9] 26546075

[B25] Sarbaz Y. Banaie M. Pooyan M. Gharibzadeh S. Towhidkhah F. Jafari A. (2012). Modeling the gait of normal and Parkinsonian persons for improving the diagnosis. Neuroscience Letters, 509(2), 72–5. [DOI:10.1016/j.neulet.2011.10.002] [PMID ] 22085691

[B26] Sarbaz Y. Gharibzadeh S. Soltanzadeh A. Towhidkhah F. (2013). A novel clinical gait-test protocol for separating parkinsonian patients from normal persons in early disease stages. Journal of Medical Imaging and Health Informatics, 3(1), 7–11. [DOI:10.1166/jmihi.2013.1125]

[B27] Sarbaz Y. Gharibzadeh S. Towhidkhah F. Banaie M. Jafari A. (2011). A gray-box neural network model of Parkinson’s disease using gait signal. Basic and Clinical Neuroscience, 2(3), 33–42.

[B28] Sarbaz Y. Towhidkhah F. Gharibzadeh S. Jafari A. (2012). Gait spectral analysis: An easy fast quantitative method for diagnosing Parkinson’s Disease. Journal of Mechanics in Medicine and Biology, 12(3), 1250041. [DOI:10.1142/S0219519411004691]

[B29] Sarbaz Y. Towhidkhah F. Jafari A. Gharibzadeh S. (2012). Do the chaotic features of gait change in Parkinson’s disease. Journal of Theoretical Biology, 307, 160–7. [DOI:10.1016/j.jtbi.2012.04.032] 22588024

[B30] Stegemöller E. L. Wilson J. P. Hazamy A. Shelley M. C. Okun M. S. Altmann L. J. (2014). Associations between cognitive and gait performance during single-and dual-task walking in people with Parkinson’s Disease. Physical Therapy, 94(6), 757–66. [DOI:10.2522/ptj.20130251] [PMID ] [PMCID ] 24557652 PMC4040423

[B31] Wild L. B. de Lima D. B. Balardin J. B. Rizzi L. Giacobbo B. L. Oliveira H. B. (2013). Characterization of cognitive and motor performance during dual-tasking in healthy older adults and patients with Parkinson’s disease. Journal of Neurology, 260(2), 580–9. [DOI:10.1007/s00415-012-6683-3] [PMID ] 23052601

[B32] Wylie S. A. van den Wildenberg W. Ridderinkhof K. R. Claassen D. O. Wooten G. F. Manning C. A. (2012). Differential susceptibility to motor impulsivity among functional subtypes of Parkinson’s disease. Journal of Neurology, Neurosurgery & Psychiatry, 83(12), 1149–54. [DOI:10.1136/jnnp-2012-303056] [PMID ] [PMCID ] PMC3704227 22917670

